# Supplementary material for: Arabidopsis γ-H2A.X-INTERACTING PROTEIN participates in DNA damage response and safeguards chromatin stability
Source: Nat Commun. 2022 Dec 26;13:7942. doi: 10.1038/s41467-022-35715-2 (PMC9792525; doi:10.1038/s41467-022-35715-2)
Supplement: Supplementary file 1 — Supplementary Information [file 41467_2022_35715_MOESM1_ESM.docx]

**
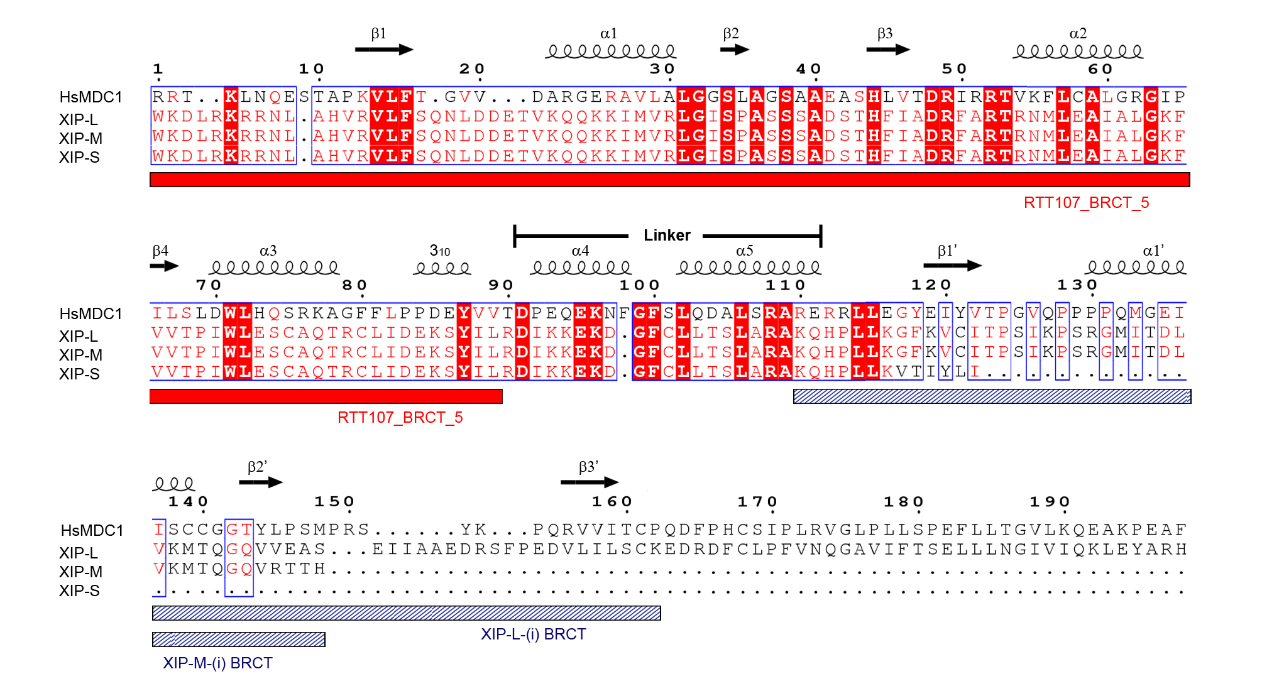
**

**Supplementary Fig. 1 Alignment of BRCT-like domain(s) of XIP isoforms and HsMDC1.**

All the encoded XIP isoforms contain a significant RTT107_BRCT_5 (PF16770) domain, and a linker composed of α4 and α5. XIP-L also harbors an additional C-terminal insignificant BRCT (PF00533) domain, and has the closest secondary structure of complete dual-BRCT-like domain to HsMDC1. The C-terminus of XIP-M extends to the second β-fold (β2’), which was thus still considered as an insignificant (i) BRCT (PF00533) domain in Prosite analysis (http://prosite.expasy.org/).

The secondary structural elements of human MDC1 (residues 1,891–2,086) (PDB: 2AZM, crystal structure of the BRCT repeat region from MDC1) were visualized together with the sequence alignment using the ESPript web server (http://espript.ibcp.fr/).

**
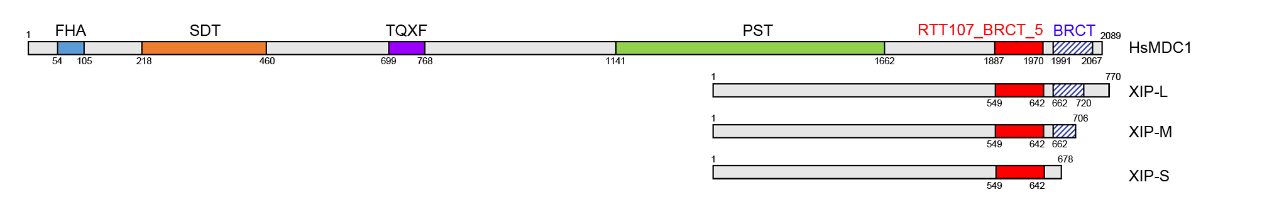
**

**Supplementary Fig. 2 XIP has little similarity with HsMDC1 except dual-BRCT-like domain**

Schematic protein structure of HsMDC1 and XIP protein isoforms. HsMDC1 is a huge protein (2089-aa-long) harboring multiple functional domains which collectively function as scaffold for diverse protein-protein interaction^1^. Except the dual-BRCT-like domain, XIP has little similarity with HsMDC1. Acronyms: Forkhead-associated (FHA) domain; Ser/Asp/Thr (SDT) region; Ser/Thr-Gln-X-Phe (TQXF) motif; Pro/Ser/Thr-rich repeat (PST) domain.


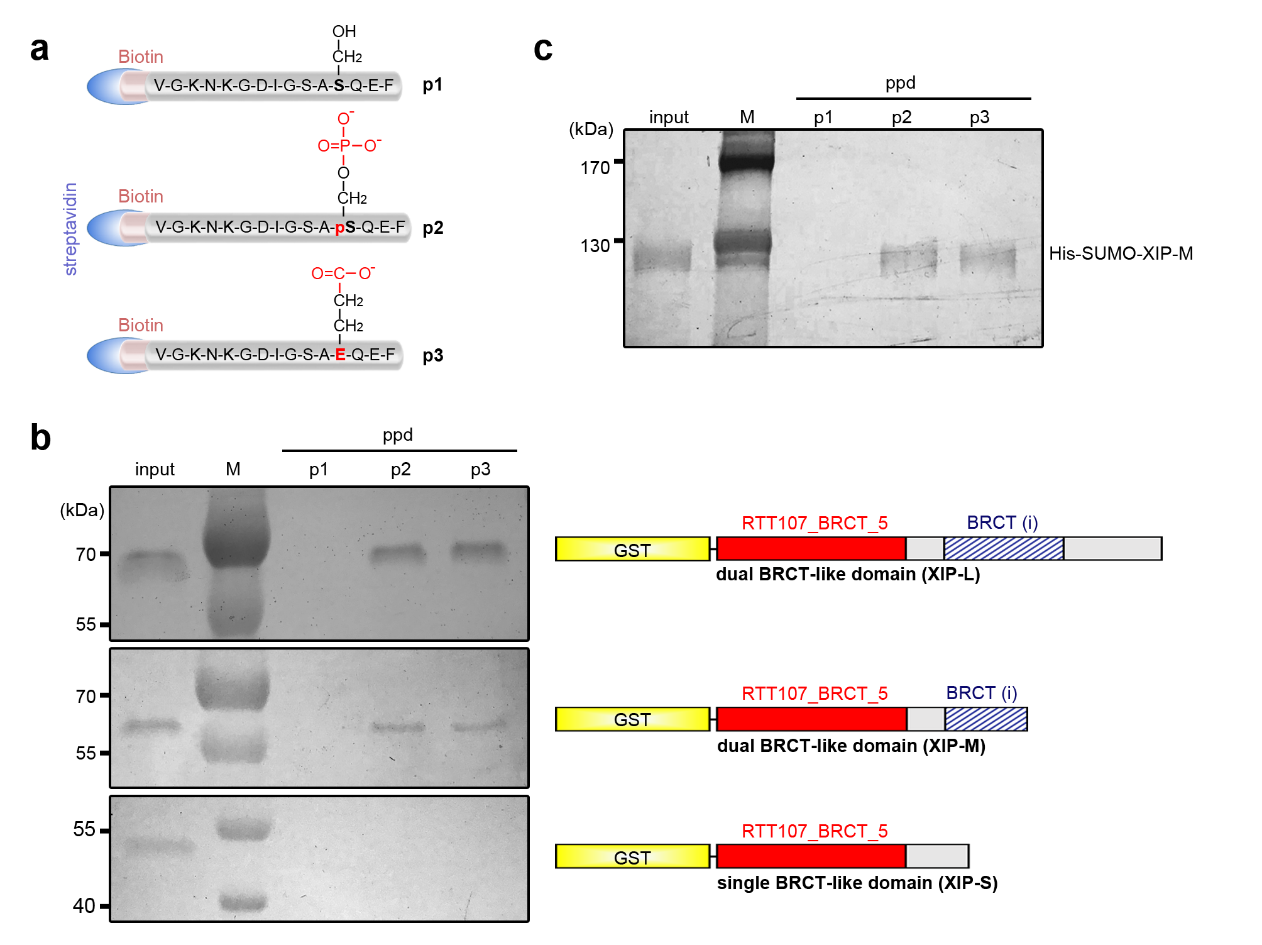


**Supplementary Fig. 3 The dual-BRCT-like domain of XIP specifically recognized γ-H2A.X.**

**a** Schematic diagram of N-terminally biotinylated peptides p1, p2 and p3.

**b** The peptide pulldown (ppd) assay by using GST-tagged dual- or single-BRCT-like domain(s) of XIP-L, XIP-M, and XIP-S, respectively. Note that single-BRCT-like domain (XIP-S) cannot be retained in ppd experiment. Representative result from two independent ppd experiments was shown.

**c** The ppd assay by using full-length XIP-M. Representative result from two independent ppd experiments was shown.

**
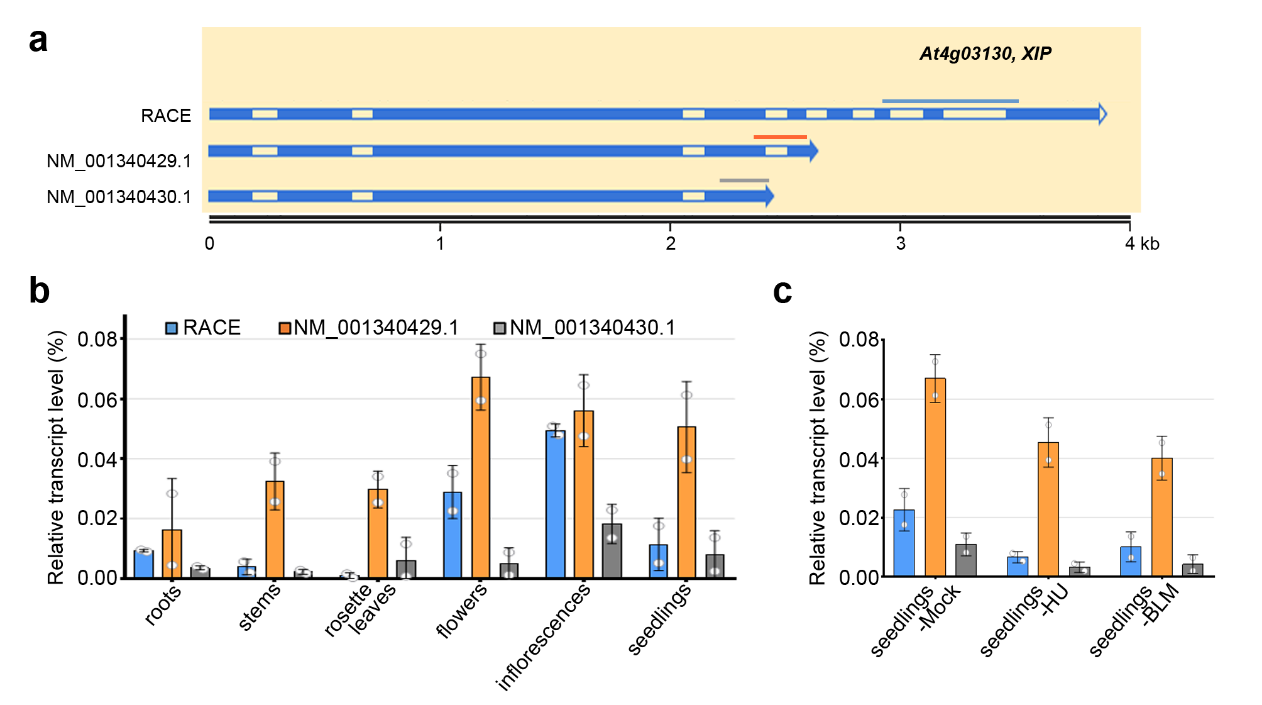
**

**Supplementary Fig. 4 The relative transcript levels of three full-length *XIP* isoforms.**

**a** Isoform-specific primers were designed to distinguish the three full-length transcripts of *XIP* in this study. Note that, in the case of RACE (blue) and NM_001340429.1 (orange), one primer in each primer pair spans an exon-exon junction to ensure the isoform-specificity. The reverse primer for NM_0001340430.1 (grey) locates in its isoform-specific terminus.

**b** Relative transcript levels of the three full-length *XIP* transcripts in different plant organs. *ACT2* was used as reference gene. Mean values were shown together with error bars indicating ±SD from two independent biological replicates.

**c** Relative transcript levels of the three full-length *XIP* transcripts in seedlings grown with or without genotoxin treatment (24-hour-long treatment of 1mM HU or 6-hour-long treatment of 2.5 μM BLM). *ACT2* was used as reference gene. Mean values were shown together with error bars indicating ±SD from two independent biological repeats.

**
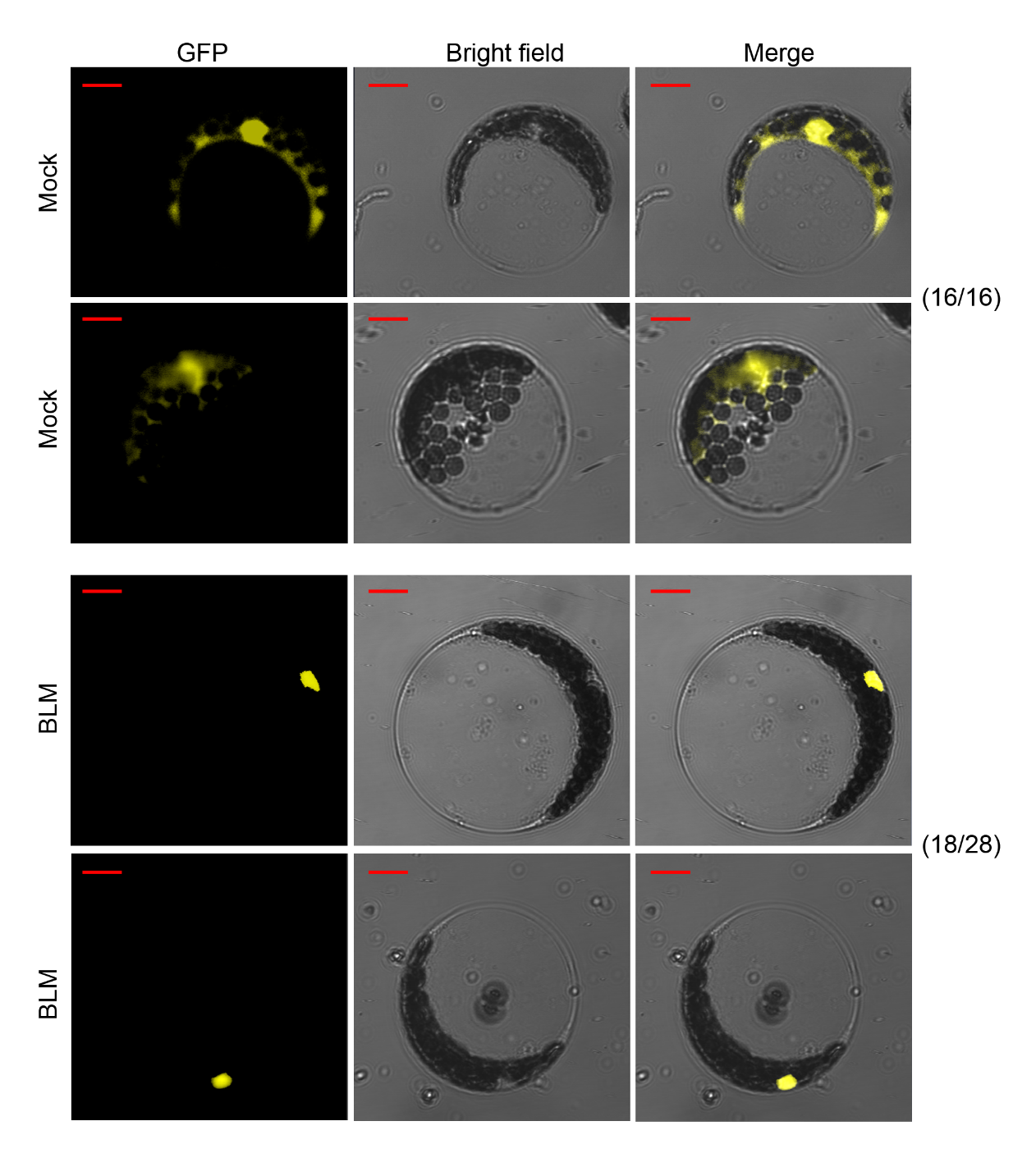
**

**Supplementary Fig. 5 The subcellular localization of YFP-XIP-M.**

The XIP-M was expressed as an YFP-fused protein in mesophyll protoplast. The transfected protoplasts were treated with or without 1 μM BLM and then were used for fluorescent observation. Note that YFP-XIP-M was localized in both cytoplasm and nuclei in all the samples under mock treatment (16 out of 16). Shortly after BLM treatment (30 min), YFP-XIP-M has been found enriched in the nuclei in most observed protoplasts (18 out of 28). Bar=10 μm.

**
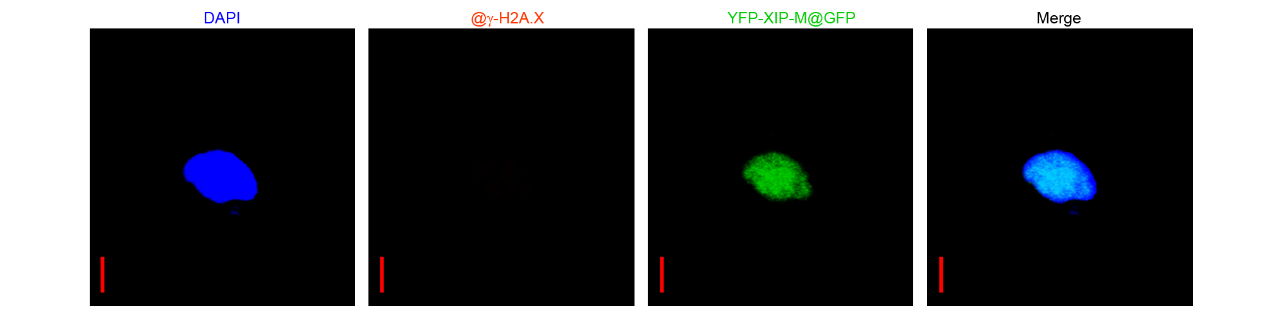
**

**Supplementary Fig. 6 The localization of YFP-XIP-M under mock condition.**

The XIP-M was expressed as an YFP-fused protein in mesophyll protoplast. The transfected protoplasts without genotoxin treatment were used as a negative control of **Fig.1e** for immuno-staining. Note that no γ-H2A.X signal was found in these protoplasts. Different from the fluorescent observation in intact cells in **Supplementary Fig.5**, the immuno-staining protocol involves membrane breakage for antibody to enter the cell as well as extensive washes. Probably for this reason, only nuclear YFP-XIP signal were clearly observed. Representative image from 8 well-stained protoplasts was shown. Bar=5 μm.

**
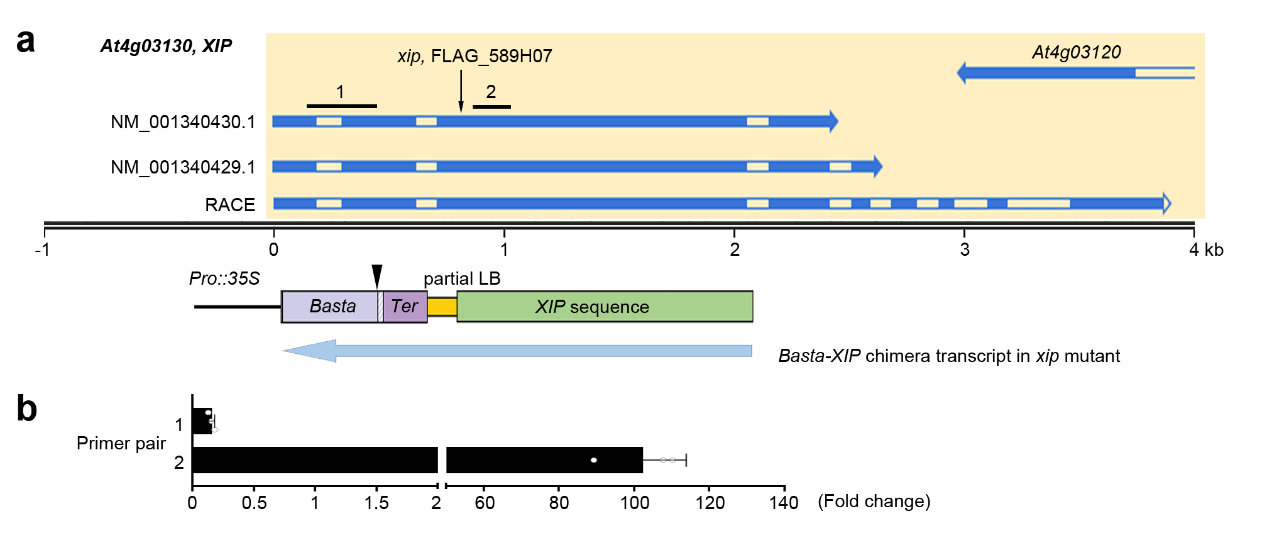
**

**Supplementary Fig. 7 Identification and characterization of *xip* mutant.**

**a** Two primer pairs (1 and 2) flanking the T-DNA insertion site were designed to examine the transcript remnant in the *xip* mutant.

**b** Primer pair 1 detected transcript in *xip* mutant at less than 10% of the WT level, while the amplicon of primer pair 2 increased dramatically to over 100-fold. *ACT2* was used as reference gene. Mean values of the relative transcript levels were shown together with error bars indicating ±SD from three independent biological replicates.

A 5'-RACE analysis was then performed to investigate the abnormal initiation site of such transcript. We found that the constitutive promoter *Pro:35S* within the integrated T-DNA sequence strongly expressed the intact resistance gene *Basta*. However, the transcript also unexpectedly included partial left border (LB) sequence and the partial *XIP* gene sequence immediately following the T-DNA insertion site, thus forming a *Basta-XIP* chimera transcript in *xip* mutant **(a)**. Since the predicted coding region of such chimera transcript does not contain any XIP protein sequence, we inferred that *xip* corresponds to a functional knock-out mutant.


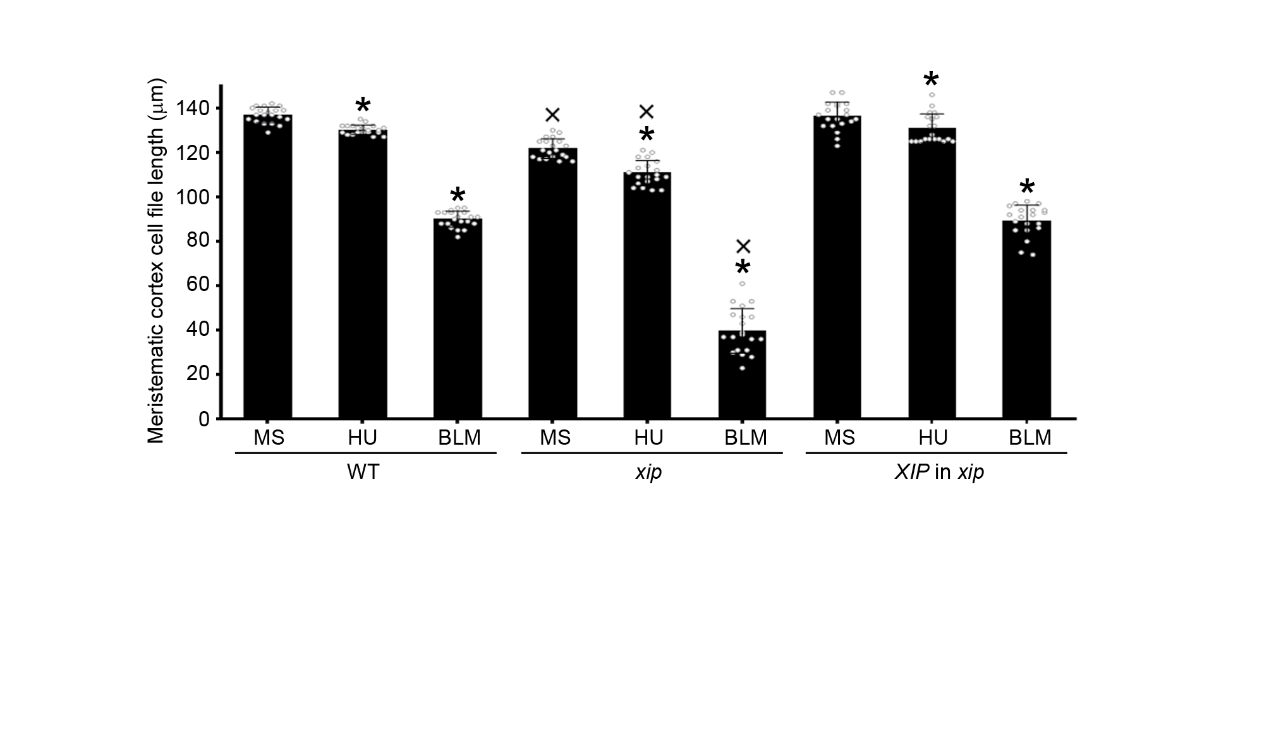


**Supplementary Fig. 8 Comparison of root meristem length.**

The meristem size was quantified by measuring the length of proximal meristem (in a cortex cell file extending from the quiescent center to the first elongated cortex cell)^2^. Mean values of 20 root tips were shown together with error bars indicating ±SD. Asterisks (*) indicate statistically significant difference (*p* < 0.05, *t*-test, two-tail) of samples treated with and without long-term HU/BLM stresses (such as WT-HU vs WT-MS, *xip*-HU vs *xip*-MS). “X” indicates statistically significant difference (*p* < 0.05, *t*-test, two-tail) in *xip* when compared with WT sample grown under the same conditions (such as *xip*-MS vs WT-MS, *xip*-HU vs WT-HU). All the *p* values can be found in the source data.

**
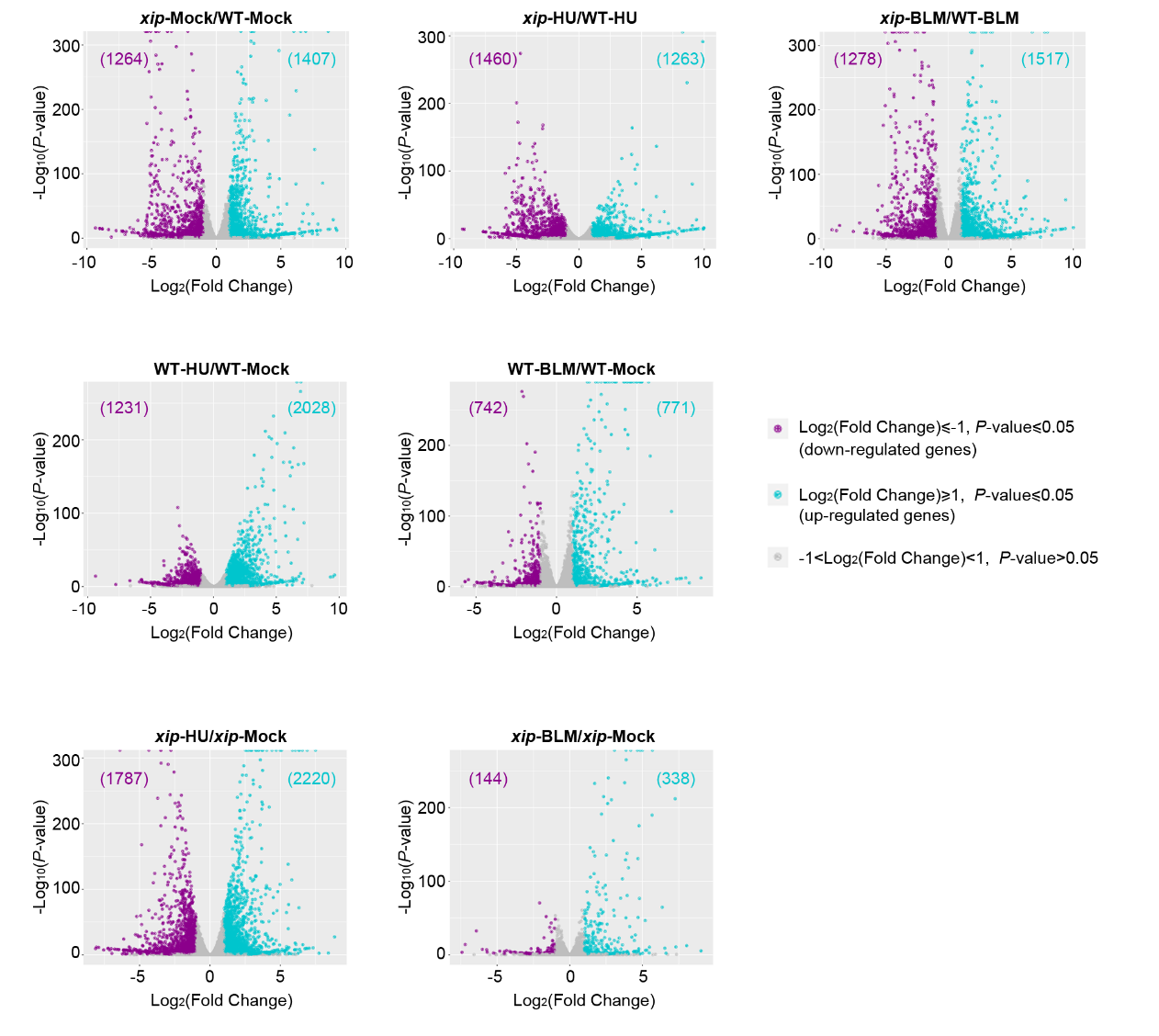
**

**Supplementary Fig. 9 Massive transcriptional changes in *XIP*-dependent and/or genotoxin-dependent manner.**

Volcano plot showing the differentially expressed genes (DEGs) in different sample pairs (*xip* vs WT, HU vs Mock and BLM vs Mock). The x-axis indicates the fold change of transcript level, and the y-axis indicates the significance scores. The *p* values were calculated and adjusted with the Benjamini-Hochberg correction. Cyan and purple points represented the genes exhibiting significantly up- and downregulated genes (fold change≥2, *p*-value≤0.05, two-tail), respectively.


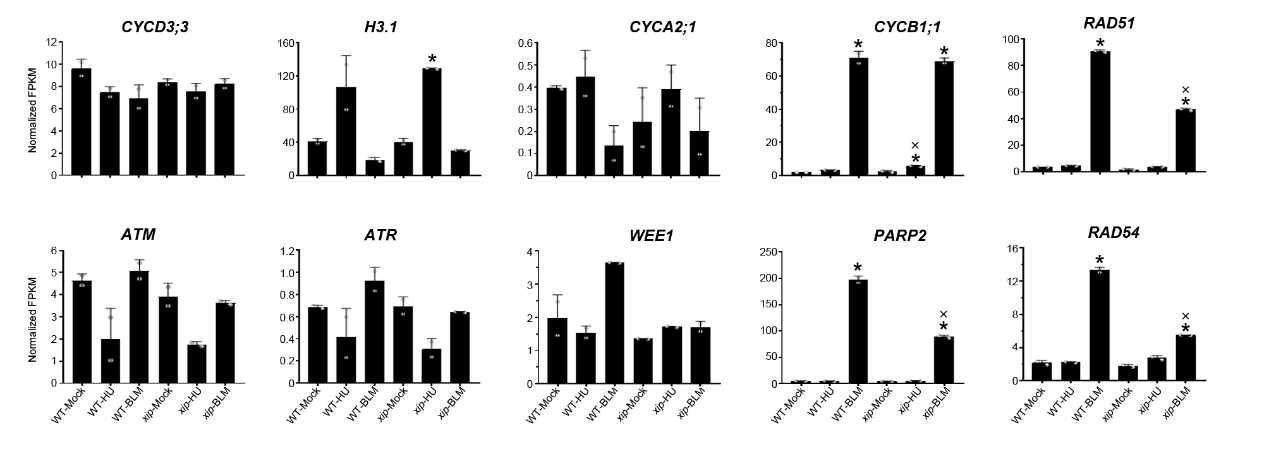


**Supplementary Fig. 10 The normalized FPKM of selected genes in this study.**

The normalized Fragment Per Kilobase of transcript per Million fragments mapped (FPKM) of all the examined genes in Fig.3a and 3b were extracted from RNA-seq with two independent biological replicates. Mean values were shown together with error bars indicating ±SD. Asterisks (*) indicate both statistically significant difference (*p* < 0.05, *t*-test, two-tail) and fold change > 2 in samples when compared with WT-Mock. “X” indicates statistically significant difference (*p* < 0.05, *t*-test, two-tail) in *xip* samples when compared with WT sample under the same conditions (such as *xip*-HU vs WT-HU, *xip-*BLM vs WT-BLM). All the *p* values can be found in the source data.

**
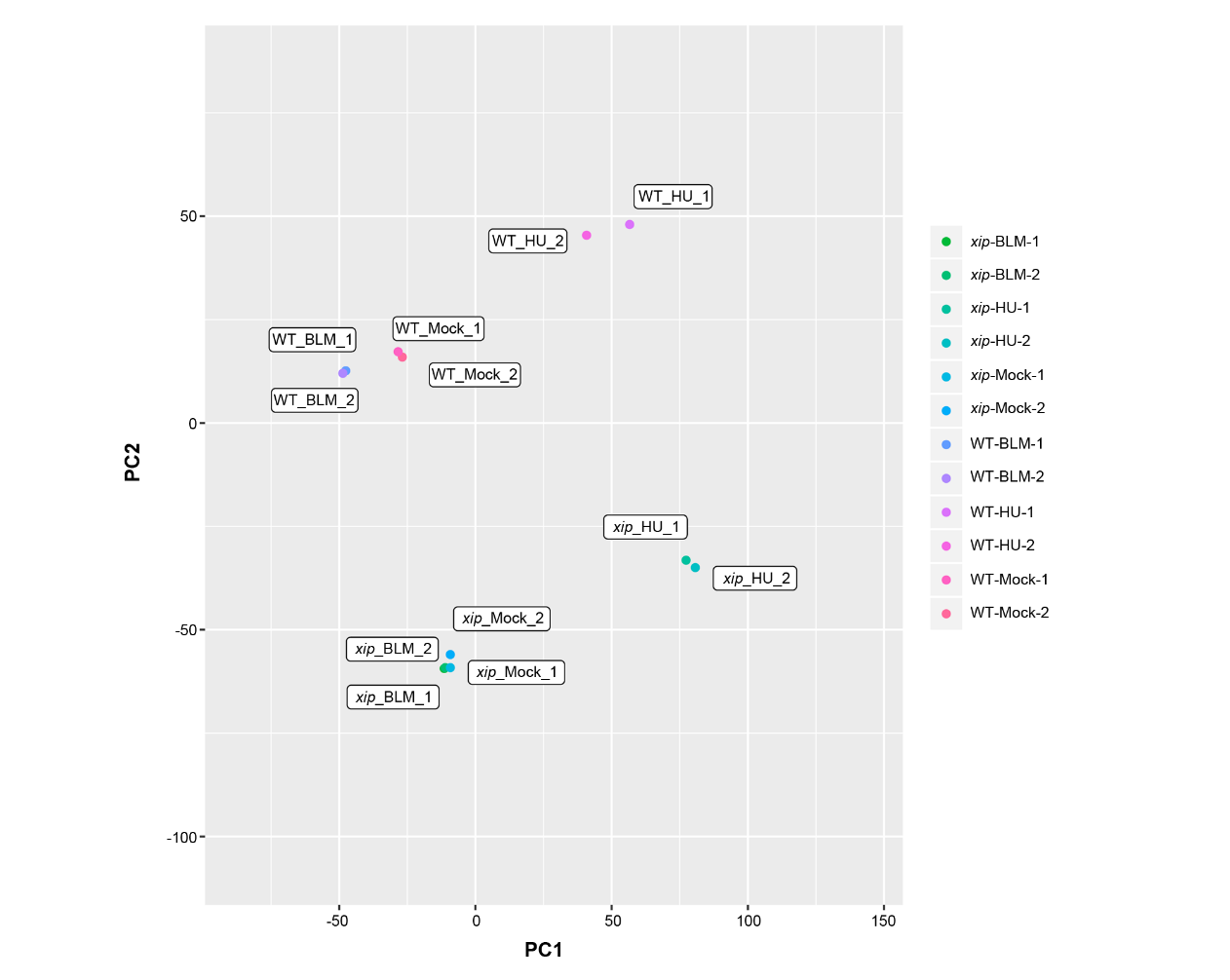
**

**Supplementary Fig. 11 The principal component analysis (PCA) of WT and *xip* samples in this study.**

The 6 samples in two biological repeats was analyzed in PCA. Note that the *xip*-Mock samples are much closer to *xip*-BLM samples in PCA analysis when compared to their WT counterparts (WT-Mock vs WT-BLM).

**
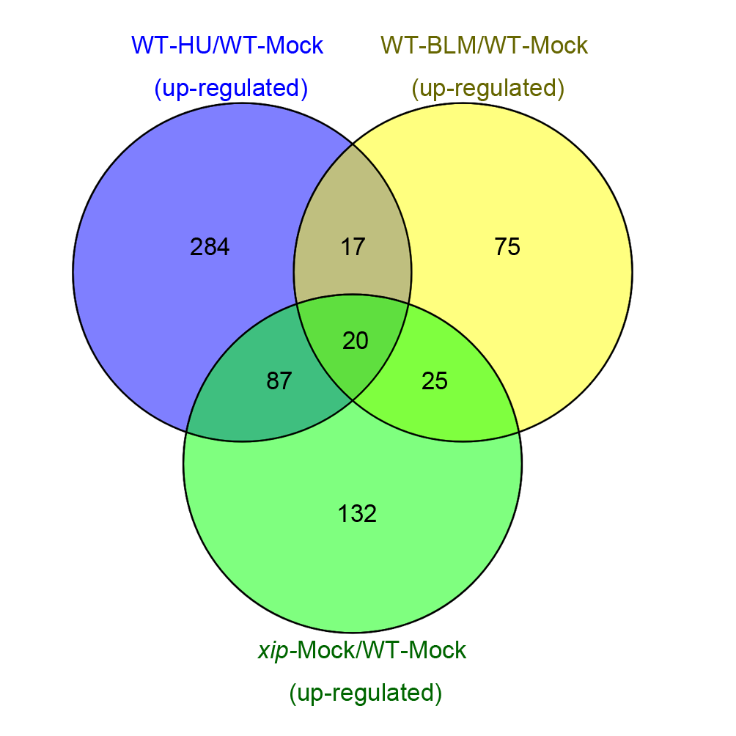
**

**Supplementary Fig. 12 *XIP*-depletion can partially mimic the plants under replication and/or genotoxin stresses in activating responsive genes.**

Venn diagram for the overlap of upregulated DEGs in enriched GO category ‘response to stress (GO: 0006950)’ in *xip* mutant (*xip-*Mock/WT-Mock) with those in WT-HU/WT-Mock and WT-BLM/WT-Mock.

**
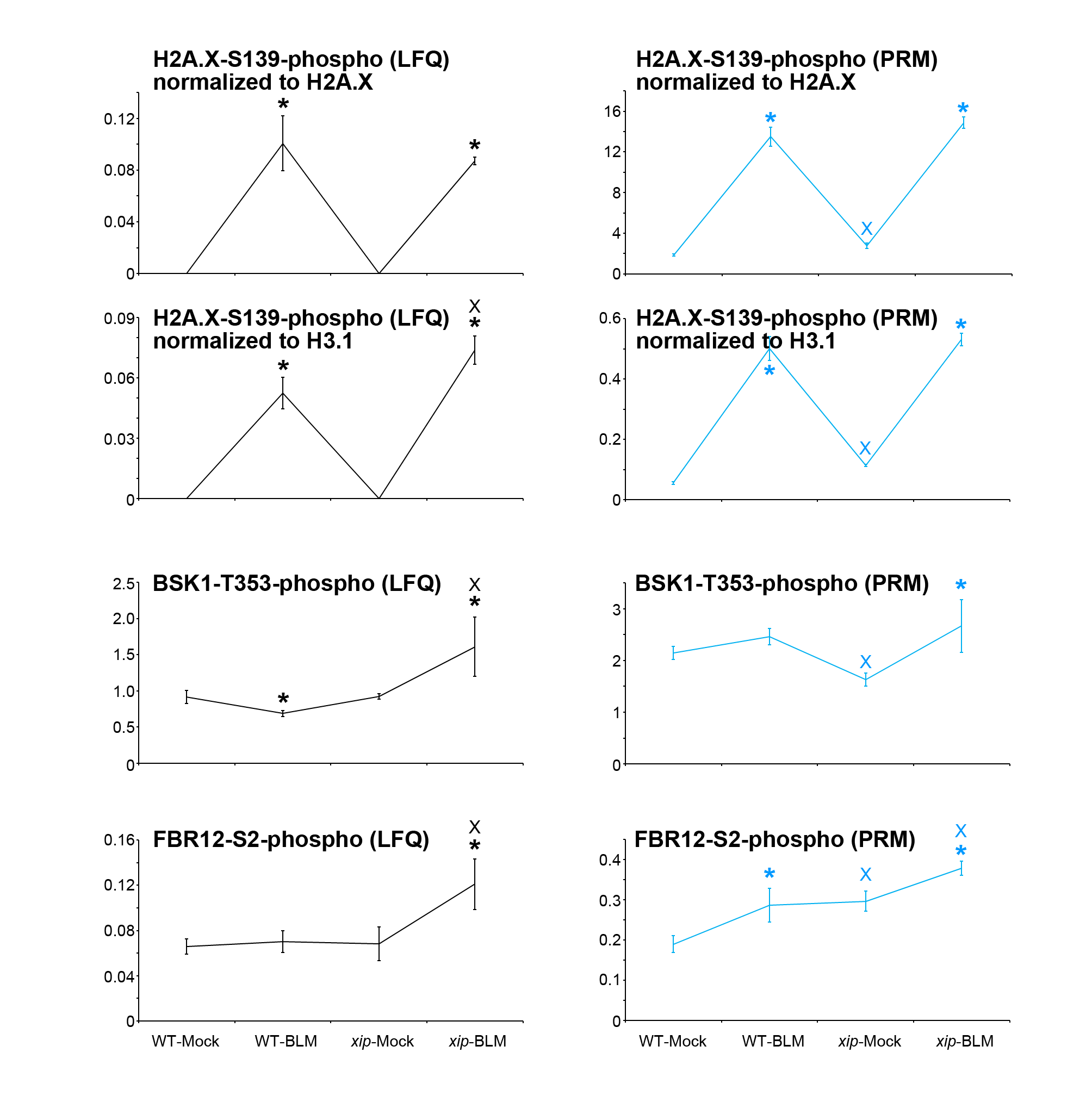
**

**Supplementary Fig. 13 The comparison of phosphorylation site of selected target proteins identified by label-free quantification (LFQ) and parallel reaction monitoring (PRM).**

Typical phospho-proteins extracted from our LFQ and PRM phospho-proteomics. The abundance of phospho-peptide was normalized to the abundance of corresponding protein. The average of these normalized values from three biological repeats is shown together with error bars indicating ±SD. Asterisks (*) indicate the statistically significant differences of samples after 6 hours of 2.5 μM BLM treatment when compared to Mock (WT-BLM vs WT-Mock, *xip*-BLM vs *xip*-Mock, *p* < 0.05, *t*-test, two-tail). “X” indicates statistically significant differences in *xip* when compared to WT (*xip*-Mock vs WT-Mock, *xip*-BLM vs WT-BLM, *p* < 0.05, *t*-test, two-tail). All the *p* values can be found in the source data.

The selected DPPs include the S139 residue in H2A.X; the T353 residue in receptor-like cytoplasmic kinase Brassinosteroid-signaling kinase 1 (BSK1), which is implicated in cell cycle progression^3^; and S2 residue of Fumonisin B1-resistant 12 (FBR12), which is implicated in cell death^4^. Notably, H2A.X-S139 phosphorylation level was also normalized to canonical histone H3.1, since the relative γ-H2A.X level was normalized to H3 in histone Western (**Fig. 4a**).

**
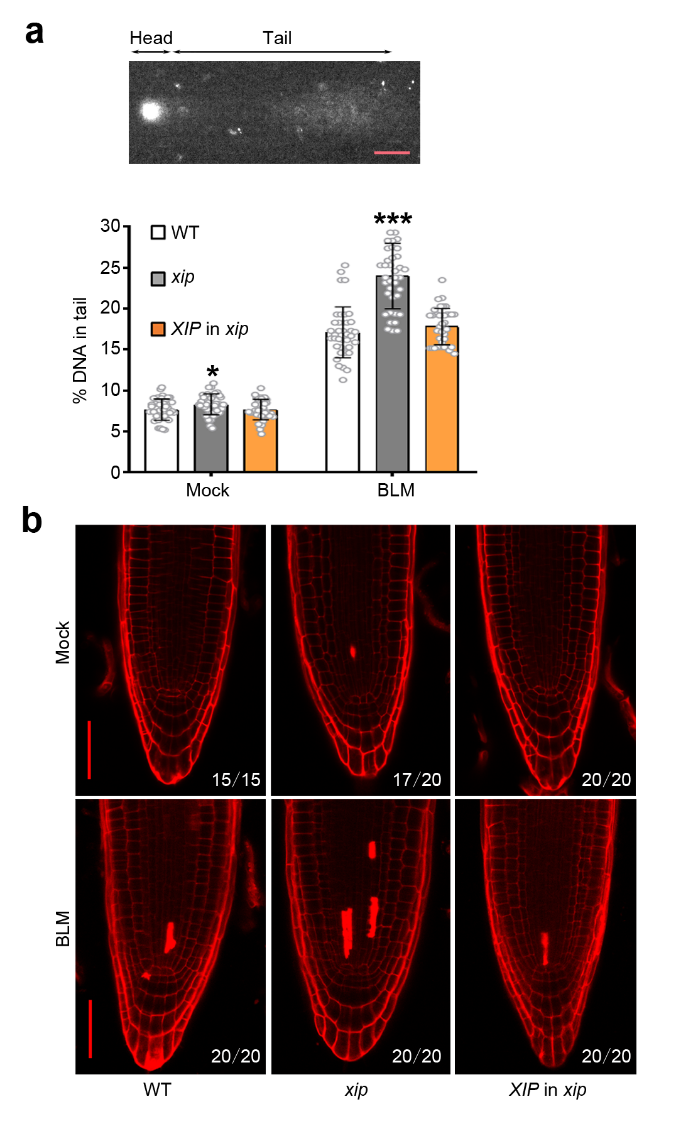
**

**Supplementary Fig. 14 The higher chromatin instability caused by *XIP* deficiency.**

**a.** Comparison of nuclear DNA lesion levels. The upper image illustrates the DNA lesion measurement in the comet assay. Bar= 10 μm. The bottom graph shows the percentage of DNA in the comet tail in the 12-day-old WT, *xip* and *XIP* in *xip* seedlings with or without 6 hours of 2.5 μM BLM treatment. For each plant sample, the mean value of 40 nuclei was shown together with error bar indicating ±SD from three independent biological replicates. Asterisks indicated the significant differences between the WT and *xip* mutant (****p*≤0.001, * *p*<0.05, *t*-test, two-tail). All the *p* values can be found in the source data.

**b.** Representative image of 15-20 PI-stained root tips of 6-days-old WT, *xip* and *XIP* in *xip* seedlings with or without 6 hours of 2.5 μM BLM treatment. Bar = 50 μm.


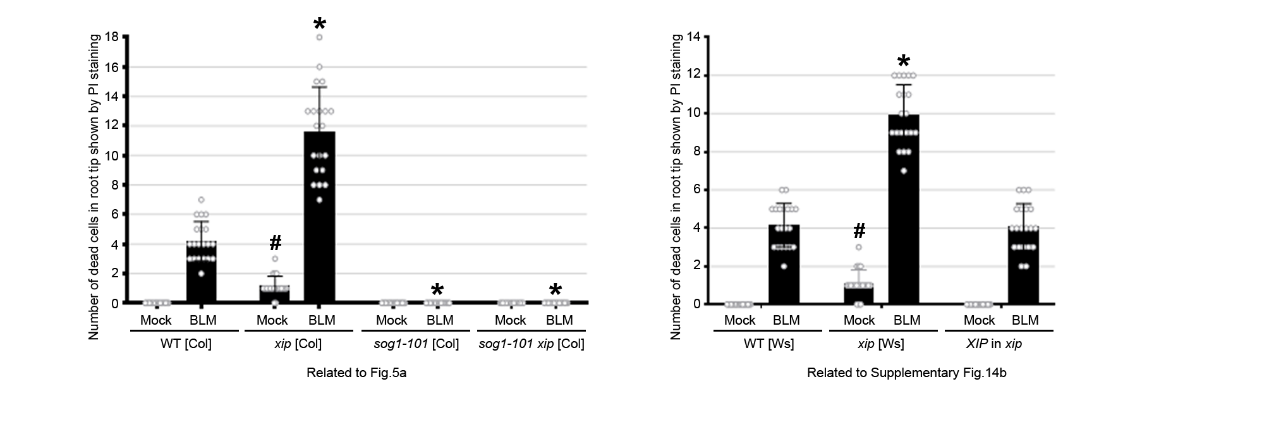


**Supplementary Fig. 15 The number of dead cells in root tips.**

The number of dead cells in each root tip (**left**, related to **Fig.5a**; **right**, related to **Supplementary Fig.14b**) was counted. The mean value was shown together with error bar indicating ±SD from 20 biologically independent plants. No dead cell was found in some background, such as *sog1-101*. In these cases, we thus only examined 15 plant samples. Pound signs (#) indicate the significant differences of samples under mock condition with WT under mock condition (WT-Mock) (*p*<0.05, *t*-test, two-tail). Asterisks (*) indicated the significant differences of samples after BLM treatment with WT after BLM treatment (WT-BLM) (*p*<0.05, *t*-test, two-tail). All the *p* values can be found in the source data.


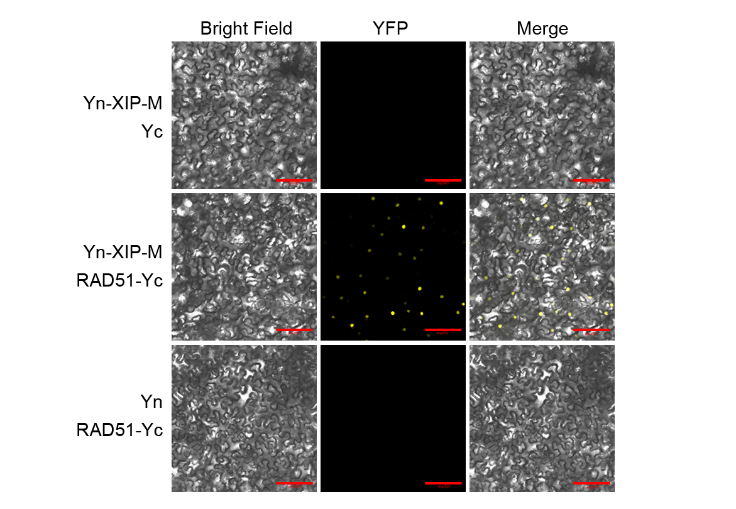


**Supplementary Fig. 16 BiFC analysis of interaction between XIP-M and RAD51.**

XIP-M and RAD51 were fused the N-terminal and C-terminal of YFP (Yn-XIP-M and RAD51-Yc), respectively, and formed visible YFP fluorescence signal in tobacco leaf cells. In contrast, no YFP signal was detected in the negative controls. Representative results from 10 leaves were shown. Bar=100 μm.


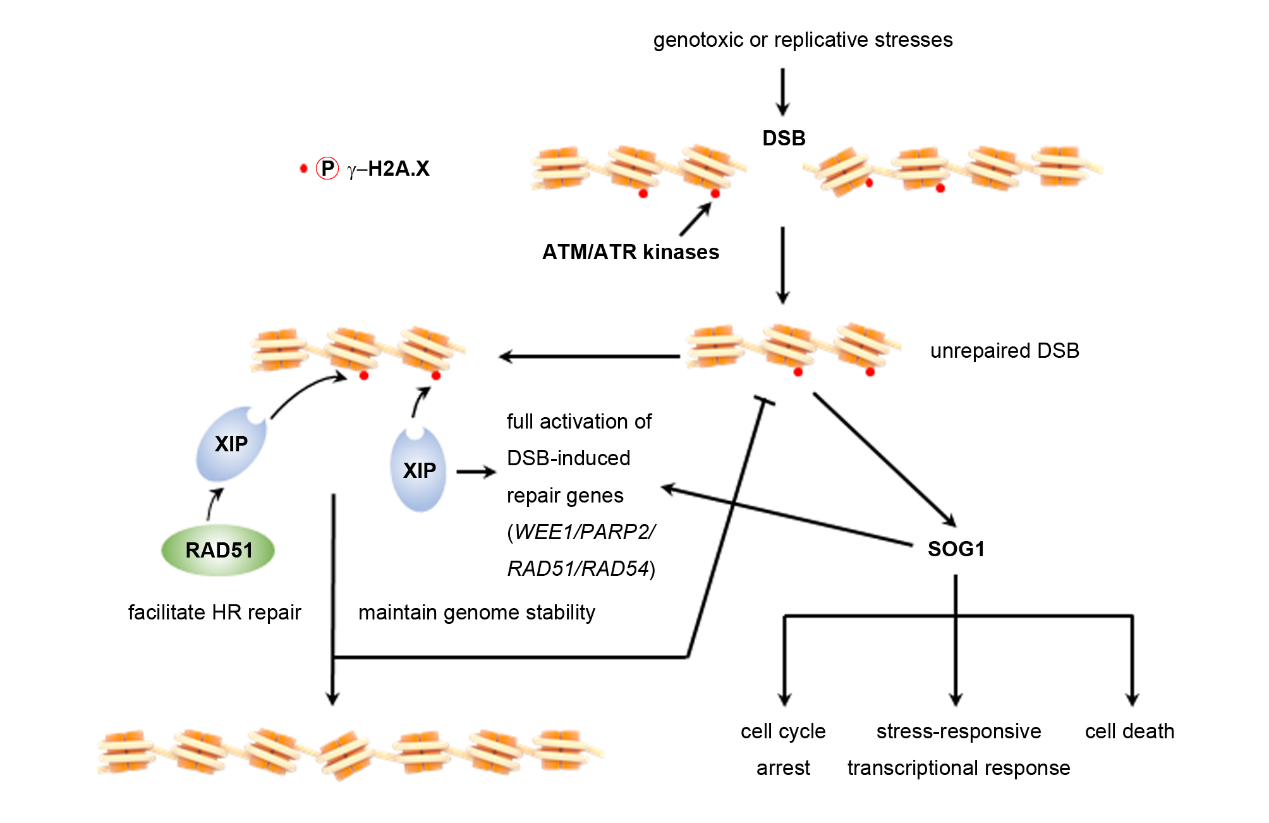


**Supplementary Fig. 17 A model of XIP activity in plant DSB response.**

XIP specifically interacts with DSB-induced γ-H2A.X, and recruits RAD51 through protein-protein interaction to facilitate HR repair and maintain genome stability.

**Supplementary References**

1. Jungmichel S, Stucki M. MDC1: The art of keeping things in focus. *Chromosoma* **119**, 337-349 (2010).

2. Perilli S, Sabatini S. Analysis of root meristem size development. *Methods Mol Biol* **655**, 177-187 (2010).

3. Yan H, Zhao Y, Shi H, Li J, Wang Y, Tang D. BRASSINOSTEROID-SIGNALING KINASE1 Phosphorylates MAPKKK5 to Regulate Immunity in Arabidopsis. *Plant Physiol* **176**, 2991-3002 (2018).

4. Feng H, Chen Q, Feng J, Zhang J, Yang X, Zuo J. Functional characterization of the Arabidopsis eukaryotic translation initiation factor 5A-2 that plays a crucial role in plant growth and development by regulating cell division, cell growth, and cell death. *Plant Physiol* **144**, 1531-1545 (2007).
