# Supplementary material for: Arabidopsis γ-H2A.X-INTERACTING PROTEIN participates in DNA damage response and safeguards chromatin stability
Source: Nat Commun. 2022 Dec 26;13:7942. doi: 10.1038/s41467-022-35715-2 (PMC9792525; doi:10.1038/s41467-022-35715-2)
Supplement: Supplementary file 3 — Description of Additional Supplementary Files [file 41467_2022_35715_MOESM3_ESM.pdf]

Description of Additional Supplementary Files:

Supplementary Data 1: The retrieved proteins by p1 and p2 in ppd-MS analysis.

Supplementary Data 2: The differentially expressed genes (DEGs) revealed in the RNA-seq in this study.

Supplementary Data 3: All the phospho-peptides identified in the phospho-proteomic analysis in this study.

Supplementary Data 4: All the proteins (for normalization) identified in the phospho-proteomic analysis in this study.

Supplementary Data 5: Normalization of phospho-peptides to the corresponding proteins, and the DPPs identified in this study.

Supplementary Data 6: PRM verification of phosphorylation site of selected target proteins in this study.

Supplementary Data 7: The XIPIPs identified in the pulldown-MS analysis in this study.

Supplementary Data 8: The primers used in this study.
